# Public health risks of approving drugs for the treatment of childhood obesity in Brazil

**DOI:** 10.1590/0102-311XEN031624

**Published:** 2024-09-20

**Authors:** Márcia Regina Vítolo, Paola Seffrin Baratto, Sophie Deram

**Affiliations:** 1 Universidade Federal de Ciências da Saúde de Porto Alegre, Porto Alegre, Brasil.; 2 Faculdade de Medicina, Universidade de São Paulo, São Paulo, Brasil.

Recent approvals by the Brazilian Health Regulatory Agency (Anvisa, acronym in Portuguese) of treatment drugs that simulate the actions of the GLP-1 peptide in the body (liraglutide and semaglutide) for weight loss in adolescents (12 years of age and older) brings questions about the relevance and risks of using these drugs to treat childhood obesity. This argument is founded on five considerations: (1) the expansion of these treatment drugs for use in adolescents was based only on randomized clinical studies developed by the pharmaceutical industry that markets them; (2) the severity of side effects; (3) the absence of studies that evaluate long-term impacts; (4) the strategies to combat obesity in Brazil and internationally, and (5) alternatives to medicalization by investments in social justice.

Use of GLP-1 agonists for treating adolescent obesity in Brazil began with the approval for liraglutide administration in August 2020, followed by the expanded use of semaglutide in September 2023. Such approval is founded on the publication of two randomized clinical studies developed by the pharmaceutical company Novo Nordisk, the first with liraglutide ^1^ and second with semaglutide ^2^. However, it is widely known that several scientific studies are needed before gathering sufficient evidence to support public recommendations or guidelines. Approval of these two drugs in Brazil for treating obesity in adolescents (12 years of age and older) has no robust evidence of efficacy, no study of effectiveness and a complete lack of knowledge regarding long-term effects, as well as presents conflict of interest. For example, the study on semaglutide ^2^ for adolescents was based on a methodology used for adults ^3^. However, the study with adults concluded with 81% of the initial sample of 1,961 participants after 68 weeks, whereas the study with adolescents concluded with 196 participants from the initial 201 after 68 weeks. Thus, this second research included a population group of lesser representativeness and greater biological vulnerability, involving different pubertal stages and peculiarities between sexes. Additionally, the results registered on the ClinicalTrials platform (https://clinicaltrials.gov/) revealed that about 36% of body weight loss in adults ^3^ was due to the loss of muscle mass; not such data is available for the adolescent sample ^2^. Conducting studies with children and adolescents poses a huge challenge for researchers as metabolic diversity and intense changes in biology and body composition limit the research results. Hence the following questions: what are the metabolic consequences of muscle mass loss in growing individuals? What are the emotional and psychological consequences for children and adolescents of aggressive treatments such as the use of medication?

A recent review ^4^ on the common adverse effects of GLP-1 receptor agonists in children and adolescents revealed events of vomiting, nausea, diarrhea, constipation, abdominal distension, abdominal pain, headache, dizziness, fatigue, dyspepsia, eructation, hypoglycemia in patients with type 2 diabetes mellitus, gastroenteritis, flatulence, gastroesophageal reflux disease, thyroid C-cell tumors, acute pancreatitis, sudden gallbladder disease, hypoglycemia, sudden kidney injury, diabetic retinopathy in individuals with type 2 diabetes mellitus, and suicidal ideation and behavior. Another publication highlights concerns regarding the late impact on child growth and development, medication abuse among patients with eating disorders or in certain sports practices, excessive or insufficient medical prescription in populations with a high prevalence of obesity and low physical fitness ^5^.

Importantly, the use of drug therapies that simulate the role of incretins in metabolism was primarily developed for treating type 2 diabetes mellitus, whose clinical conditions have serious consequences for patients’ general health and a high risk of mortality. However, we must evaluate the cost-benefit of its widespread use for weight loss. Wilding et al. ^6^ observed that patients regained two-thirds of the weight lost after stopping taking the medication, subliminally suggesting that use of this drug should be continuous and probably lifelong since obesity is a chronic disease and therefore requires chronic treatment ^6^.

GLP-1 is a hormone released in the intestine during food digestion that acts on pancreatic beta cells by increasing insulin production, inhibiting glucagon secretion, delaying gastric emptying and decreasing appetite. Another potential action found in a study was the increase and proliferation of pancreatic beta cells in young animals, but not adults, suggesting a difference in physiological impact in growing individuals ^7^. Additionally, GLP-1 receptors are present in other tissues including the thyroid, exocrine pancreas, meninges, renal tubules, bones, and their activation promote changes unrelated to glucose homeostasis. Hence the enormous concern about the widespread use of its agonists for weight loss in adolescents. Endogenous GLP-1 has a short half-life (< 2 minutes), is transiently taken up by its receptors and strongly regulated under healthy physiological conditions; however, these treatment drugs abruptly alter normal physiology, extending the time of action of GLP-1 receptors (approximately one week). Besides, there is insufficient evidence on long-term health damage. Interestingly, Butler et al. ^8^ state that the biggest issue would not be pancreatitis, which has been identified in the use of GLP-1 agonist medications such as liraglutide, but rather the probable proliferation of pancreatic ducts, acinar metaplasia and subclinical pancreatitis. Clinical pancreatitis is only the tip of the iceberg. Periductal alpha cell hyperplasia can cause obstruction and evolve to neuroendocrine neoplasia.

Another systematic review ^9^ concluded that the use of GLP-1 agonist drugs was effective, safe and acceptable for weight reduction and glucose control in children and adolescents with obesity. However, great caution is needed in considering this systematic review as a reliable evidence parameter. It included six clinical trials with liraglutide, five of which had conflict of interest (funded by Novo Nordisk) and one study did not disclose the presence or absence of a conflict of interest. As for semaglutide, the only clinical trial reviewed was developed by Novo Nordisk. We therefore consider it premature to conduct systematic reviews to evaluate the efficacy of these medications in children and adolescents before we have studies without conflict of interest and researchers without ties to the pharmaceutical industry.

The World Health Organization ^10^ included childhood obesity as one of its priorities to establish strategies to combat it, considering that 41 million children under 5 are overweight. In Brazil, national data from the *Brazilian Household Budgets Survey* (POF, acronym in Portuguese) of 2008-2009 ^11^ showed an overweight prevalence of 33.5% in children aged 5-9 years, in line with *Brazilian National Survey on Child Nutrition* (ENANI, acronym in Portuguese) data from 2019 ^12^ which showed an overweight (body mass index > 1 Z-score) prevalence of 28.3% in children under 5 years of age. In a longitudinal analysis, data from the Pelotas cohort revealed an 88% increase in the prevalence of children with overweight aged 12 months between 1982 and 2015 ^13^. These numbers show that childhood obesity has intensified in Brazil and is occurring at an increasingly early age. Effective strategies should therefore be established to prevent the occurrence of obesity during the window of opportunity that is the first 1,000 days of life ^14^.

Recently, a successful experience in implementing actions to promote healthy eating in the first two years of life has shown a relevant impact on reducing childhood adiposity at 6 years of age in Southern Brazil ^15^. At the national level, since 2021 the Strategy for Prevention and Care of Childhood Obesity (Proteja, acronym in Portuguese), aims to halt the advance of childhood obesity and improve children’s health and nutrition by actions such as food and nutrition surveillance, health promotion in schools, continuous training of professionals involved in child care and intersectoral articulations for promoting healthy environments within cities.

Also, the American Academy of Pediatrics published guidelines for the clinical practice of childhood obesity treatment which emphasized the use of medications and bariatric surgery and paid little attention to behavioral changes such as diet and exercise ^16^. In response to this new paradigm, Ludwig & Holst ^17^ published an opinion against the medicalization of childhood obesity, advocating greater investment in effective strategies related to diets and lifestyle - which they called social justice. The authors argue that focusing on weight loss, as is the case with the use of these medications, does not necessarily improve the health of children and adolescents who, with lower weight but still engaging in unhealthy eating habits and sedentary lifestyle, will not be protected from cardiovascular diseases, cancer and other chronic diseases.

In time, we must add the high treatment cost in Brazil to the disadvantages and risks of using injectable medications for treating obesity in children and adolescents. Currently, the price of injectable treatment drugs can vary between Brazilian pharmacies as long as they do not exceed the Maximum Consumer Price (PMC, acronym in Portuguese) established by Anvisa. In practice, a single dose of injectables can equal the value of a minimum wage for a month’s formal work, which makes access to the medicine unviable for the most vulnerable populations and too costly for the federal government to subsidize.

In conclusion, the use of GLP-1 receptor agonists such as semaglutide, liraglutide and, in the future, tirzepatide (recently registered with Anvisa) in light of current evidence, lead to proven side effects that compromise quality of life during treatment and later, due to the unknown long-term effects of these innovative drug therapies on the physiological and clinical conditions of this vulnerable population group.
