# Supplementary material for: Public health risks of approving drugs for the treatment of childhood obesity in Brazil
Source: Cad Saude Publica. 2024 Sep 20;40(9):e00031624. doi: 10.1590/0102-311XEN031624 (PMC11415047; doi:10.1590/0102-311XEN031624)
Supplement: Translation

# Translation: Riscos para a saúde pública da aprovação de medicamentos para o tratamento da obesidade infantil no Brasil

**DOI:** 10.1590/0102-311XPT031624

**Authors:** Márcia Regina Vítolo, Paola Seffrin Baratto, Sophie Deram

**Affiliations:** 1 Universidade Federal de Ciências da Saúde de Porto Alegre, Porto Alegre, Brasil.; 2 Faculdade de Medicina, Universidade de São Paulo, São Paulo, Brasil.

Diante das recentes aprovações pela Agência Nacional de Vigilância Sanitária (Anvisa) do uso de medicamentos que simulam as ações do peptídeo GLP-1 no organismo (liraglutida e semaglutida) para perda de peso em adolescentes a partir de 12 anos de idade, questiona-se a pertinência e os riscos do uso desses medicamentos no tratamento da obesidade infantil. A motivação para essa argumentação baseia-se em cinco considerações: (1) a ampliação do uso desses medicamentos para adolescentes foi fundamentada apenas nos estudos clínicos randomizados desenvolvidos pela própria indústria farmacêutica que os comercializa; (2) a gravidade dos efeitos colaterais; (3) a ausência de estudos que avaliam o impacto a longo prazo; (4) estratégias de combate à obesidade no Brasil e no mundo; e (5) alternativas à medicalização por meio de investimento em justiça social.

O uso de agonistas de GLP-1 para o tratamento da obesidade em adolescentes no Brasil teve início com a aprovação do uso da liraglutida, em agosto de 2020, seguida da ampliação do uso da semaglutida, em setembro de 2023. A origem das liberações para adolescentes se dá pelas publicações de dois estudos clínicos randomizados desenvolvidos pela indústria farmacêutica Novo Nordisk, sendo o primeiro com a liraglutida ^1^ e o segundo com a semaglutida ^2^. É de amplo conhecimento que são necessários vários estudos científicos antes de se considerar que existem evidências suficientes para embasar recomendações ou diretrizes no âmbito público. A aprovação do uso desses dois medicamentos no Brasil para o tratamento de obesidade em adolescentes a partir dos 12 anos não tem evidência robusta da eficácia, não apresenta estudo de efetividade e há total desconhecimento dos efeitos a longo prazo, além da presença de conflito de interesses. Como exemplo, o estudo da semaglutida ^2^ para adolescentes a partir de 12 anos foi baseado na metodologia utilizada para adultos ^3^; contudo, no estudo com adultos, o número amostral inicial foi de 1.961 e concluído com 81% da amostra nas 68 semanas da pesquisa, enquanto no estudo com adolescentes, o número amostral foi de 201 e concluído com 196 participantes nas 68 semanas de estudo. Assim, o estudo com adolescentes incluiu um grupo populacional de menor representatividade e maior vulnerabilidade biológica, que envolve diferentes estágios pubertários e peculiaridades entre os sexos. Além disso, no estudo com adultos ^3^, os resultados registrados na plataforma ClinicalTrials (https://clinicaltrials.gov/) revelaram que cerca de 36% da perda de peso corporal foi proveniente da perda de massa muscular; no estudo com adolescentes, esses dados não estão disponíveis ^2^. A realização de estudos com crianças e adolescentes é um enorme desafio para pesquisadores, porque a diversidade metabólica e as mudanças intensas na biologia e na composição corporal limitam os resultados dos estudos. Nesse sentido, questiona-se: quais as consequências metabólicas da perda de massa muscular em indivíduos em crescimento? Quais as consequências emocionais e psicológicas de tratamentos agressivos como o uso de medicamentos para crianças e adolescentes?

Recente revisão ^4^ sobre os efeitos adversos comuns dos agonistas do receptor de GLP-1 em crianças e adolescentes revelou a presença de: vômito, náusea, diarreia, prisão de ventre, distensão abdominal, dor abdominal, dor de cabeça, tontura, fadiga, dispepsia, eructação, hipoglicemia em pacientes com diabetes mellitus tipo 2, gastroenterite, flatulência, doença do refluxo gastroesofágico, tumores de células C da tiroide, pancreatite aguda, doença súbita da vesícula biliar, hipoglicemia, lesão renal súbita, retinopatia diabética em indivíduos com diabetes mellitus tipo 2 e ideação e comportamento suicida. Outra publicação destaca preocupações em relação ao impacto tardio no crescimento e desenvolvimento infantil, ao abuso do medicamento entre pacientes com transtornos alimentares ou em determinadas práticas esportivas, à prescrição médica excessiva ou insuficiente em populações com alta prevalência de obesidade e baixo condicionamento físico ^5^.

Destaca-se ainda que o uso de terapias medicamentosas que simulam o papel das incretinas no metabolismo foi primariamente desenvolvido para o tratamento do diabetes mellitus tipo 2, cujas condições clínicas remetem a consequências graves na saúde geral dos pacientes, além do alto risco de mortalidade. Entretanto, é necessário avaliar o custo-benefício de sua utilização generalizada para a perda de peso, já que no estudo de Wilding et al. ^6^ os pacientes ganharam dois terços do peso perdido após a interrupção do uso da medicação. Assim, os autores sugerem, subliminarmente, que o uso desse medicamento deve ser contínuo e provavelmente para toda a vida, uma vez que a obesidade é uma doença crônica e, portanto, requer tratamento crônico ^6^.

Sabe-se que GLP-1 é um hormônio liberado no intestino durante a digestão dos alimentos e que age nas células beta do pâncreas aumentando a produção de insulina, inibindo a secreção de glucagon, atrasando o esvaziamento gástrico e diminuindo o apetite. Outra ação potencial foi o aumento e proliferação das células beta do pâncreas em animais jovens, mas não adultos, o que sugere diferença de impacto fisiológico em indivíduos em crescimento ^7^. Além disso, os receptores para GLP-1 estão presentes em outros tecidos, incluindo tireoide, pâncreas exócrino, meninges, túbulos renais, ossos, e sua ativação vai promover mudanças não relacionadas à homeostase da glicose. Por esse motivo, dá-se a enorme preocupação com o uso generalizado dos seus agonistas para perda de peso entre adolescentes. Fisiologicamente, o GLP-1 endógeno tem meia vida curta (< 2 minutos), é captado pelos seus receptores de forma transitória e fortemente regulada em condições fisiológicas saudáveis. Porém, esses medicamentos alteram bruscamente a fisiologia normal, ampliando o tempo de ação dos receptores para a GLP-1 (aproximadamente 1 semana), e não há evidências suficientes dos prejuízos para a saúde a longo prazo. Posicionamento interessante de Butler et al. ^8^ coloca que o maior problema não seria a pancreatite que foi identificada no uso de medicamentos agonistas da GLP-1, como a liraglutida, mas sim a provável proliferação dos ductos pancreáticos, metaplasia acinar e pancreatite subclínica. A pancreatite clínica é a ponta do iceberg. A hiperplasia das células alfa periductais podem causar obstrução e progressão para neoplasia neuroendócrina.

Outra revisão sistemática ^9^ concluiu que o uso de medicamentos agonistas da GLP-1 foi efetivo, seguro e aceitável para a redução de peso e controle da glicose em crianças e adolescentes com obesidade. Entretanto, é necessário ter muita cautela para considerar essa revisão sistemática um parâmetro de evidência confiável. A revisão incluiu seis ensaios clínicos com liraglutida, sendo cinco com conflito de interesses (financiados pela indústria farmacêutica Novo Nordisk) e um estudo que não revelou a presença ou não de conflito de interesses. Para a semaglutida, o único ensaio clínico que entrou nessa revisão foi o desenvolvido pela Novo Nordisk. Assim, consideramos prematura a realização de revisões sistemáticas para avaliar a eficácia desses medicamentos em crianças e adolescentes antes que tenham estudos isentos de conflito de interesses e de pesquisadores sem vínculos com a indústria farmacêutica.

A Organização Mundial da Saúde ^10^ incluiu a obesidade infantil como uma das prioridades para estabelecer estratégias de combate, considerando que 41 milhões de crianças menores de cinco anos apresentam excesso de peso. No Brasil, dados nacionais da *Pesquisa de Orçamentos Familiares* (POF) de 2008-2009 ^11^ mostraram prevalência de excesso de peso de 33,5% em crianças de 5-9 anos, em consonância com os dados do *Estudo Nacional de Alimentação e Nutrição Infantil* (ENANI) de 2019 ^12^ que mostraram prevalência de excesso de peso (escore Z do índice de massa corporal > 1) de 28,3% em menores de cinco anos. Em análise longitudinal, dados da coorte de Pelotas revelaram que, entre 1982 e 2015, houve aumento de 88% na prevalência de excesso de peso das crianças com 12 meses de idade ^13^. Esses números mostram que a obesidade infantil se agravou no Brasil e vem ocorrendo cada vez mais precocemente. Dessa forma, é necessário estabelecer estratégias efetivas para prevenir a ocorrência da obesidade durante a janela de oportunidade que se estabelece nos primeiros mil dias de vida ^14^.

Recentemente, a experiência exitosa em implementar ações para promoção de alimentação saudável nos primeiros dois anos de vida mostrou impacto relevante na redução da adiposidade infantil na idade de seis anos no sul do Brasil ^15^. No âmbito nacional, desde 2021, a Estratégia de Prevenção e Atenção à Obesidade Infantil (Proteja) tem como objetivo deter o avanço da obesidade infantil e contribuir para a melhoria da saúde e da nutrição das crianças por meio de ações, como a vigilância alimentar e nutricional, promoção de saúde nas escolas, educação permanente de profissionais envolvidos no cuidado às crianças e articulações intersetoriais para a promoção de ambientes saudáveis no âmbito das cidades.

Há pouco tempo, a Academia Americana de Pediatria publicou as diretrizes para a prática clínica do tratamento da obesidade infantil, nas quais enfatizou a utilização de medicamentos e cirurgia bariátrica e deu pouca atenção às mudanças comportamentais, como alimentação e atividade física ^16^. Em resposta a esse novo paradigma, Ludwig & Holst ^17^ publicaram opinião contrária à medicalização da obesidade infantil, defendendo maior investimento em estratégias eficazes relacionadas a dietas e estilo de vida, o que chamaram de justiça social. Os autores sugeriram que focar na perda de peso, como é o caso do uso dessas medicações, não necessariamente impacta em melhor saúde das crianças e adolescentes, que, com menor peso, mas ainda com práticas alimentares não saudáveis e sedentarismo, não estarão livres de doenças cardiovasculares, câncer e outras doenças crônicas.

Em tempo, é necessário somar o elevado custo do tratamento no Brasil às desvantagens e riscos de se utilizar medicações injetáveis para o tratamento da obesidade em crianças e adolescentes. Atualmente, o preço dos medicamentos injetáveis pode variar entre farmácias brasileiras, desde que não extrapole o Preço Máximo ao Consumidor (PMC) estabelecido pela Anvisa. Na prática, uma única dose dos injetáveis pode se igualar ao valor de um salário-mínimo referente a um mês de trabalho formal, o que faz com que o acesso ao medicamento seja inviável às populações mais vulneráveis e demasiadamente oneroso para que seja subsidiado pelo Governo Federal.

Em conclusão, enfatizamos que o uso de agonistas do receptor de GLP-1, como a semaglutida, liraglutida e, futuramente, a tirzepatida (recentemente registrada na Anvisa), à luz das evidências atuais, acarretam efeitos colaterais já comprovados que comprometem a qualidade de vida durante o tratamento e posteriormente, pelos efeitos de longo prazo desconhecidos dessas inovadoras terapias medicamentosas nas condições fisiológicas e clínicas desse vulnerável grupo populacional.
